# Supporting data for the photo-induced deformation behavior for AZO-containing polymers connected by hydrogen bonding

**DOI:** 10.1016/j.dib.2019.104849

**Published:** 2019-11-21

**Authors:** Yihan Wang, Lizhi Hu, Qiang Yin, Kai Du, Taoran Zhang, Qinjian Yin

**Affiliations:** a College of Chemistry, Sichuan University, Chengdu, 610064, China; b Research Center of Laser Fusion, China Academy of Engineering Physics, P.O. Box 919-987, Mianyang, 621900, China

**Keywords:** Photo-induced deformation, AZO-containing polymers, Hydrogen bonding

## Abstract

Besides covalent bond [1], the non-covalent interactions are expected to drive the photo-induced behavior. The data presented here in this article shows the effect of the azobenzene ratio to photo-induced deformation behaviors of aggregates self-assembled from poly (styrene-*stat*-4-vinylpyridine) (PS-*stat*-P4VP) and azobenzene (AZO) 4-phenylazophenol, which are connected with hydrogen bonding interaction, confirmed by ^13^C-NMR spectra and FTIR spectra. The average major-to-minor axis ratio (*l/d*) (*l* represent long axis of ellipsoid and *d* represent minor axis) could reveal the deformation degree of the aggregates [2]. The synthesis process of PS-*stat*-P4VP/AZO was based on Wang et al. [3].

**Table:** Specifications Table

Subject area	Chemistry
More specific subject area	Self-assembly of amphiphilic polymers, photo responsive
Type of data	^13^C-NMR spectra, FTIR spectra, TEM image
How data was acquired	^13^C NMR were recorded by a Bruker AV II-400 NMR spectrometer at room temperature. FTIR measurements were conducted on a Bruker Tensor 27 spectrometer to confirm the formation of hydrogen bonding. The microstructure of the samples was examined by scanning electron microscopy (SEM, Hitachi S-4800, Japan). A polarized beam from a linearly polarized Ar^+^ laser beam (MDL-III-405-100Mw) with a wavelength of 405 nm was conducted to investigate photo-induced deformation behavior.
Data format	Raw and Analyzed
Experimental factors	AZO ratio to PS-*stat*-P4VP are changed.
Experimental features	The relationship between AZO ratio and photo-induced deformation behavior of PS-*stat*-P4VP/AZO aggregates were determined.
Data source location	Chengdu, China
Data accessibility	Data is with this article.
Related research article	Y H. Wang, L Z. Hu, Q. Yin, K Du, T R. Zhang, Q J. Yin, Multi-responsive hollow nanospheres self-assembly by amphiphilic random copolymer and azobenzene, Polymer 175, 2019, 235–242 [3].

**Table:** 

**Value of the data** • The hydrogen bonding can serve as driving force for the photo-induced deformation of AZO-polymeric assemblies. • This following data helps in defining the formation of hydrogen bonds between polymer and AZO molecules. • The comparison with various AZO ratio to polymers can help in understanding the role of azobenzene molecules on the photo-induced deformation behavior of AZO-containing polymers.

## 1 Data

**Fig. 1:**
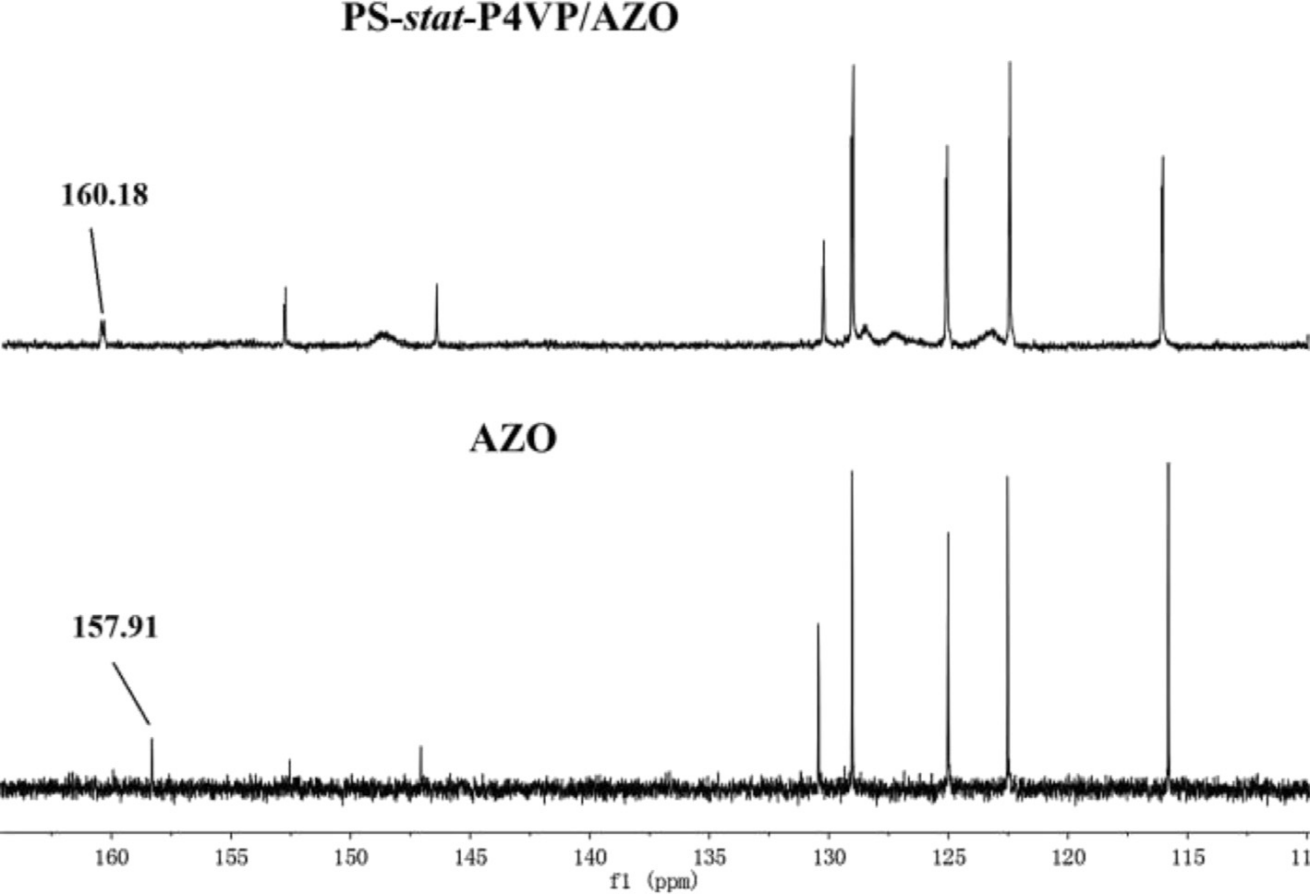
^13^C NMR results of AZO and PS-*stat*-P4VP/AZO_0.5_ in CDCl_3_.

**Fig. 2:**
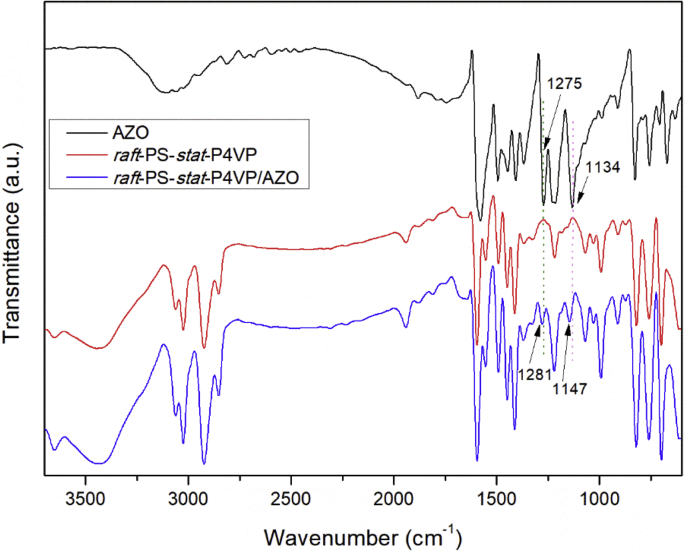
FTIR spectra of *raft*-PS-*stat*-P4VP, AZO and *raft*-PS-*stat*-P4VP/AZO hollow nanospheres.

**Fig. 3:**
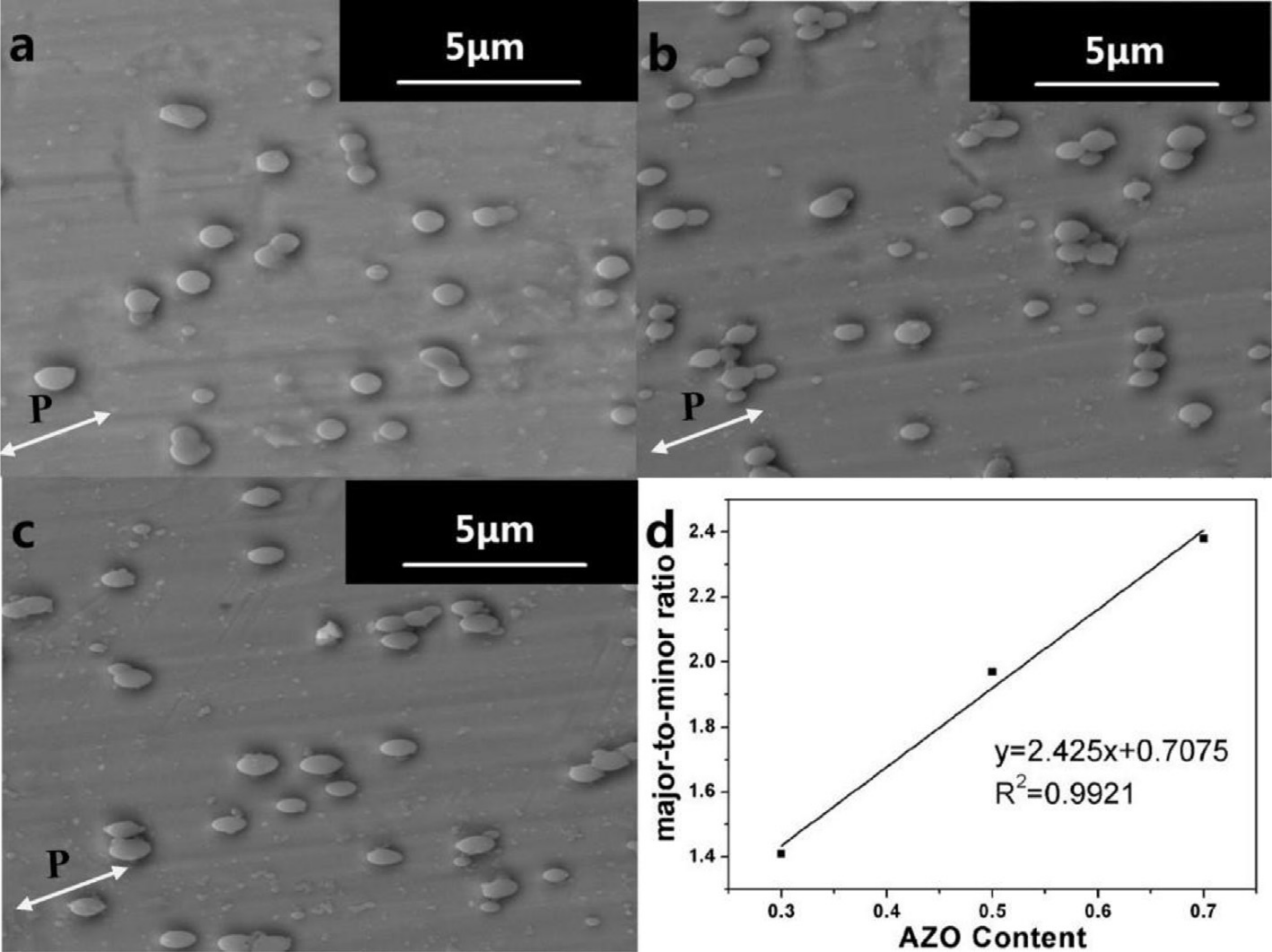
SEM images of PS-*stat*-P4VP/AZO after irradiation for different 4-VP/AZO ratio: a) 1:0.3; b) 1:0.5; c) 1:0.7; and d): relationship between the average major-to-minor ratio of PS-*stat*-P4VP/AZO and AZO content.

Here, we provided ^13^C-NMR spectra and FTIR spectrum of AZO and PS-*stat*-P4VP/AZO aggregates in Fig. 1, Fig. 2 to conform the formation of hydrogen bonds between the phenolic hydroxyl groups of AZO and the pyridine groups of PS-stat-P4VP. The relationship between the average major-to-minor axis ratio (l/d) of PS-*stat*-P4VP/AZO aggregates and AZO content are shown in Fig. 3.

The formation of the hydrogen bonds is supported by ^13^CNMR spectra as shown in Fig. 1. After introducing PS-*stat*-P4VP solution (50 vol% ethanol solution) into AZO solution, the signal of the phenolic hydroxyl carbon of AZO shifts from 157.91 to 160.18 ppm, illustrating the carboxyl hydroxyl group and the pyridyl group form into intermolecular hydrogen bonds [1]. The disappeared peak at 157.91 ppm reflects that AZO molecules have completely connected with the PS-*stat*-P4VP chains.

Fig. 2 shows the typical FTIR spectra of *raft*-PS-*stat*-P4VP, AZO and *raft*-PS-*stat*-P4VP/AZO complexes. For *raft*-PS-*stat*-P4VP, the main absorption bands of pyridine ring in P4VP appear at 1556 cm^−1^ (C=N stretching vibration) and 1413 cm^−1^ (C=C stretching vibration). Which can be ascribed to the C=N and C=C stretching vibration of pyridine ring. In the spectra of AZO, several absorption peaks are observed, including 3100 cm^−1^ (hydrogen bonds formed by phenolic hydroxyl groups of AZO), 1134 cm^−1^ (C–O stretching vibration), and 1275 cm^−1^ (O–H out-of-plane vibration of phenolic hydroxyl groups) [4]. Noting that after introducing AZO to PS-*stat*-P4VP, the characteristic absorption bands of AZO at 1134 cm^−1^ and 1275 cm^−1^ appear in *raft*-PS-*stat*-P4VP/AZO, and blue shift to 1147 cm^−1^ and 1281 cm^−1^ in *raft*-PS-*stat*-P4VP/AZO respectively, indicating the formation of hydrogen bonds between the –OH group of AZO and 4-VP.

The PS-*stat*-P4VP/AZO aggregates show “spindle-like” particles because they can be significantly elongated along the polarization direction of the polarizer. As shown in Fig. 3, after 1h irradiation, the values of *l/d* for PS-*stat*-P4VP/AZO_0.3_ is 1.51, for PS-*stat*-P4VP/AZO_0.5_ is 2.38, and for PS-*stat*-P4VP/AZO_0.7_ is 2.86. The increasement of the l/d value suggests that the azobenzene chromophores content has a significant influence on the photo-induced deformation behavior of PS-*stat*-P4VP/AZO [2].

## 2 Experimental design, materials and methods

### 2.1 Synthesis of PS-*stat*-P4VP/AZO

The materials and the synthesis of PS-*stat*-P4VP/AZO were decribed in the related research article [3]. RAFT copolymerization of 4VP (8.0 mL, 80.7 mmol), styrene (8.2 mL, 76.5 mmol) and CDB (0.2209 g, 0.812 mmol) were performed in DMF (30mL) under N_2_ atmosphere with AIBN (0.0736 g, 0.448 mmol) as initiator. The reaction was heated at 80 °C for 12h. After the system was restored to room temperature, the reaction solution was added into cold deionized water to precipitate. A pale yellow precipitate was obtained and purified by three DMF/H_2_O (v:v = 1:1) cycles. The final product was dried under vacuum at 55 °C for 5 days and labeled as PS-*stat*-P4VP. Then, PS-*stat*-P4VP was dissolved in ethanol (5mL) to prepare a solution of 0.5mg∙ml^−1^. And then mix it with AZO to prepare PS-*stat*-P4VP/AZO, as the monomer molar ratio of 4VP: AZO was 1:0.3, 1:0.5. and 1:0.7.

### 2.2 Methods

^13^C NMR were recorded by a Bruker AV II-400 NMR spectrometer at room temperature. Fourier transform infrared spectroscopy (FTIR) measurements were conducted on a Bruker Tensor 27 spectrometer to confirm the formation of hydrogen bonding. A polarized beam from a linearly polarized Ar^+^ laser beam (MDL-III-405-100Mw) with a wavelength of 405 nm was conducted to investigate photo-induced deformation behavior. The microstructure of the samples was examined by scanning electron microscopy (SEM, Hitachi S-4800, Japan).
